# A three-dimensional image processing program for accurate, rapid, and semi-automated segmentation of neuronal somata with dense neurite outgrowth

**DOI:** 10.3389/fnana.2015.00087

**Published:** 2015-07-20

**Authors:** James D. Ross, D. Kacy Cullen, James P. Harris, Michelle C. LaPlaca, Stephen P. DeWeerth

**Affiliations:** ^1^Coulter Department of Biomedical Engineering, Georgia Institute of Technology/EmoryAtlanta, GA, USA; ^2^School of Electrical and Computer Engineering, Georgia Institute of TechnologyAtlanta, GA, USA; ^3^Department of Neurosurgery, University of PennsylvaniaPhiladelphia, PA, USA; ^4^Philadelphia Veterans Affairs Medical CenterPhiladelphia, PA, USA

**Keywords:** image processing, segmentation, fluorescent microscopy, confocal, neural constructs

## Abstract

Three-dimensional (3-D) image analysis techniques provide a powerful means to rapidly and accurately assess complex morphological and functional interactions between neural cells. Current software-based identification methods of neural cells generally fall into two applications: (1) segmentation of cell *nuclei* in high-density constructs or (2) tracing of cell *neurites* in single cell investigations. We have developed novel methodologies to permit the systematic identification of populations of neuronal somata possessing rich morphological detail and dense neurite arborization throughout thick tissue or 3-D *in vitro* constructs. The image analysis incorporates several novel automated features for the discrimination of neurites and somata by initially classifying features in 2-D and merging these classifications into 3-D objects; the 3-D reconstructions automatically identify and adjust for over and under segmentation errors. Additionally, the platform provides for software-assisted error corrections to further minimize error. These features attain very accurate cell boundary identifications to handle a wide range of morphological complexities. We validated these tools using confocal z-stacks from thick 3-D neural constructs where neuronal somata had varying degrees of neurite arborization and complexity, achieving an accuracy of ≥95%. We demonstrated the robustness of these algorithms in a more complex arena through the automated segmentation of neural cells in *ex vivo* brain slices. These novel methods surpass previous techniques by improving the robustness and accuracy by: (1) the ability to process neurites and somata, (2) bidirectional segmentation correction, and (3) validation via software-assisted user input. This 3-D image analysis platform provides valuable tools for the unbiased analysis of neural tissue or tissue surrogates within a 3-D context, appropriate for the study of multi-dimensional cell-cell and cell-extracellular matrix interactions.

## Introduction

The analysis of neural tissue or tissue surrogates is increasingly performed within a three-dimensional (3-D) context as neural engineers and neurobiologists gain an appreciation for cell-cell and cell-extracellular matrix interactions across tissue-level dimensions. The study of neural cells within 3-D environments has increased in both *in vivo* and *in vitro* applications, and has benefited from advances in image acquisition technology. For example, imaging techniques such as multiphoton microscopy permit visualization and monitoring of neural cells at increasingly deep levels within the cerebral cortex in live animals (Stosiek et al., 2003; Sullivan et al., 2005; Gobel et al., 2007), providing a vast array of temporal and 3-D spatial information. Additionally, researchers are increasingly utilizing more representative 3-D cell culture systems over traditional two-dimensional (2-D) cell cultures (Yu et al., 1999; Lin et al., 2005b; Cullen and Laplaca, 2006; Cullen et al., 2011; Hopkins et al., 2015). This 3-D context presents the advantages of traditional *in vitro* systems, while allowing investigations of cellular behavior in a more physiologically-relevant state, including cell-cell and cell-extracellular matrix interactions that may be constrained in planar cultures.

As image acquisition techniques and cell culture technology advance to permit utilization of deep (i.e., thick) 3-D environments, there is an increasing need for analysis tools to facilitate the investigation of cell morphology and function within this framework. Specifically, automated image analysis routines for rapid and accurate segmentation of fluorescently labeled neural cells and/or their processes would facilitate such studies. Problems associated with automated image analysis are amplified in neural systems, where typical analyses are confounded by issues such as diverse cellular morphologies, complex process outgrowth, and high cell densities.

Accordingly, our objective was to create a robust system that allowed for rapid and accurate analysis of multiple cellular morphologies without special or multiple staining regimes. Current automated segmentation algorithms have difficulty providing high throughput mapping of complex morphological and functional interactions. For instance, although many routines can accurately quantify nuclear (i.e., spherical) labeling in 2-D or 3-D, nuclear stains alone are inherently limiting as they omit information pertaining to such important measures as cell morphology, neurite outgrowth, and cell-cell interactions (e.g., receptor-mediated or synaptic). When labeling neuronal somata, commercial software is error-prone in the quantification of 2-D neural images as large caliber processes are routinely counted as cells (unpublished observations). Furthermore, publically available systems generally fall into one of two different camps: (1) user-driven systems (e.g., Neurolucida, StereoInvestigator), which have excellent reconstruction capabilities, but are extremely time-consuming, or (2) completely automated systems, (e.g., ImageJ routines, Image Pro Plus), which may be fast, but typically offer little user control and may be inaccurate given irregular (e.g., non-spherical), process-bearing neural cell morphologies. One recent alternative to these two categories is the FARSIGHT framework that allows for complex analysis and associative image analysis (Bjornsson et al., 2008; Luisi et al., 2011). While powerful in its capabilities, this framework requires multiple histological markers and is computationally intensive. Likewise, neural-specific image processing techniques presented in the literature tend to be along one of two different applications: (1) cell population characterization, in which the algorithms segment *nuclei* of medium to high density neural constructs—including FARSIGHT Nucleus Editor (Solórzano et al., 1999; Sarti et al., 2000; Chawla et al., 2004; Bjornsson et al., 2008; Latorre et al., 2013), or (2) single cell (or low density) reconstruction, with highly detailed traces of individual cell processes—including FARSIGHT Trace Editor (Al-Kofahi et al., 2002, 2003; Koh et al., 2002; Meijering et al., 2004; Zhang et al., 2007; Luisi et al., 2011). Of note, FARSIGHT can either count nuclei *or* trace neurites in distinct modules, but currently the framework is not robust enough to handle somata and neurites together.

Given this landscape, the goal of the current work was to fill the gap in analysis capabilities. In particular, we have devised a strategy that allows one to optimize the imaging parameters for signal acquisition for neurites, which can be done via visual inspection and the precise confocal settings are not necessary as inputs into our software. Acquiring neurites often saturates the signal for cell bodies. In contrast, if you optimize for somata, one is often left with poor or non-existent information about neurites (thus standard “auto-tuning” features omit important micro-features). Of course, this issue can be partially overcome with a severely restrictive optical thickness and z-stack increment, but this often comes at the cost of requiring enormous times for image acquisition. Moreover, our approach applies for any method or stain that simultaneously labels fine feature(s) *and* robust feature(s), thus deriving multi-faceted information. In particular, our method allows the capture of information about cell bodies without discarding (or not acquiring) information about network connectivity when captured using a single label (e.g., genetic fluorescent protein expression or other cytoplasmic fluorescent markers). This method vastly increases the throughput of 3-D work at every step of the process from labeling/staining to image acquisition through quantitative analysis. Additionally, we demonstrate that this increase in throughput does not sacrifice the accuracy or quality of acquired data. The work presented here offers researchers a simple, tunable, single channel, automated image analysis tool with user-controlled corrections that can be optimized to compensate for application-specific issues associated with analysis of 3-D neural systems. Specifically, in contrast to any other current option, the analysis tool presented here offers the ability to identify neuronal somata with dense neurite arborization within thick 3-D constructs or tissue.

We accomplish this via the modification of existing techniques as well as the development of novel features. Previous studies have addressed 2-D and 3-D *nuclear* segmentation techniques (Irinopoulou et al., 1997; Lin et al., 2005a). One particular algorithm repeatedly employed is the highly efficient watershed algorithm (Umesh Adiga, 2001; Lin et al., 2003). Despite the widespread use of this algorithm for delineating cellular objects, this technique is notorious for over-segmentation, an error that occurs when distinct nuclei are broken down into multiple components. Other investigators have reduced this problem by using *a priori* knowledge to skillfully sculpt image contours that guide object segmentation. For example, one technique used a hybrid of gradient cues and geometric distance transforms, to shape the image based on both geometric and intensity features (Lin et al., 2003). Another technique avoided region based segmentation algorithms altogether and drew lines between coupled indentations or “necklines” to split overlapping nuclei (Belien et al., 2002). While these processing methods improved segmentation results, they could not entirely prevent over-segmentation. For the remaining errors, post-processing has proven to be very effective. In particular, Lin and Adiga have demonstrated excellent results by using geometric measures (e.g., area, convexity, and texture) to control merging of neighboring segmented objects (Adiga and Chaudhuri, 2001; Lin et al., 2003, 2005a).

In addition to the classic segmentation problem, neural image processing algorithms have the issue of rapidly distinguishing somata from neurites. In our application, in which we extract the boundaries of somata from images with dense neurites, the watershed “over segmentation” of neurites becomes an asset. The spiny and dimpled projections (or recessions) of the neurites “misdirect” the watershed routine and produce heavily splintered and fractured elements. Cell bodies, on the other hand, tend to have rounder, smoother morphologies. In this regard, the segmentation of cells and neurites take on entirely different shapes and sizes, and it is on the basis of these differences that we may classify and thus remove unwanted features from the image. However, while fractured segmentation is intended for neurites, it is undesirable when it occurs in somata. We can remedy cell body over-segmentation by using 3-D context clues to identify problem areas. Specifically, we can first segment cells in each 2-D frame (z-slice at a particular *z* location), and then use information from overlapping cells in adjacent frames (the *z−1 and z+1* positions) to identify errors.

Though the 3-D context can eliminate many errors, we and others have seen that it cannot eliminate all segmentation errors (Latorre et al., 2013). Therefore, we also implemented a software-assisted correction mode to reduce the error rate further. Consequently, we have utilized a multilateral solution including somata vs. neurite segmentation, 3-D context segmentation correction, and software-assisted user validation to minimize error rate.

In this paper, we present methods to rapidly and reliably distinguish cell bodies from neurites and automatically identify segmentation errors. In addition, we provide methods for software assisted manual corrections for any remaining errors. Our overall objective was to provide a quick, reliable, and easy to use algorithm that included the ability to identify neuronal somata across 3-D tissue or tissue surrogates in z-stacks preserving morphological/connectivity data, thus improving the scope and efficiency of analyses without sacrificing accuracy. We experimentally validated these techniques using confocal z-stacks taken from thick 3-D *in vitro* neural constructs consisting of simple one channel fluorescently labeled neurons with increasing neurite outgrowth and morphological complexity. Additionally, we quantified fluorescently labeled neural cells across thick organotypic *ex vivo* brain slices. The results attained by our algorithm were statistically compared to the “gold standard,” i.e., manual human analysis. This analysis platform provides valuable methods for unbiased measurements of neural cells within a 3-D context and builds toward functional tracing of neuron-neuron interactions over 4-D.

## Materials and methods

### 3-D primary cortical neuronal cultures and organotypic brain slice cultures

All procedures involving animals conformed to guidelines set forth in the NIH Guide for the Care and Use of Laboratory Animals and were approved by the Georgia Tech Institutional Animal Care and Use Committee. All cell reagents were obtained from Invitrogen (Carlsbad, CA) or Sigma (St. Louis, MO) unless otherwise noted. Tissue was harvested from Sasco Sprague-Dawley rats (Charles River, Wilmington, MA) following anesthetization using isoflurane.

Neurons were derived from embryonic day 17–18 rat fetuses by isolating the cerebral cortices, which were dissociated using trypsin (0.25%) + 1 mM EDTA (10 min at 37°C) followed by DNase (0.15 mg/mL). Neurons were entrapped in several formulations of 3-D culture matrices of Matrigel (Matrigel is primarily collagen and laminin; 7.5 mg/mL; BD Biosciences) or SeaPrep agarose (1.5%; Cambrex), either with or without collagen IV covalently crosslinked (0.3–0.6 mg/mL), as previously described (Cullen and Laplaca, 2006; Cullen et al., 2007). The cultures were 500–1000 μm thick at a final cell density of 3750–5000 cells/mm^3^, were fed neuronal medium (Neurobasal medium + 2% B-27 + 500 μM L-glutamine) and maintained in a tissue culture incubator (37°C, 5% CO_2_, 95% humidified air).

Brain slices were acquired from postnatal day 11–12 rat pups by isolating the brain and generating 400 μm thick coronal slices using a McIIwain Tissue Chopper (Mickle Laboratory Engineering, United Kingdom). The media for the first 2 days was 50% Opti-Mem + 25% Hanks Balanced Salt Solution + 25% heat inactivated horse serum + 5 mg/mL D-glucose + 1 mM L-glutamine; thereafter the media was Neurobasal medium + 2% B-27 + 5 mg/mL D-glucose + 1 mM L-glutamine. These organotypic brain slice cultures were maintained in a tissue culture incubator on Millipore membranes within 6-well plates containing 1.2 mL media per well.

### Fluorescent labeling and image acquisition

Cells and brain slices were labeled using fluorescent probes for distinguishing live and dead cells (LIVE/DEAD Viability/Cytotoxicity Kit; Molecular Probes, Eugene, OR). Cell cultures and brain slices were rinsed in buffer and incubated with 2 μM calcein AM and 4 μM ethidium homodimer-1 at 37°C for 30 min and rinsed in PBS. After viability/cytotoxicity staining, cells/slices were viewed using a Laser Scanning Confocal Microscope (Zeiss 510, Oberkochen, Germany) with Argon and Helium-Neon lasers. Images were taken at 20× and 40× magnification with a Zeiss LD Plan Apochromat 20× 0.80 N.A. with a field of view of 460.7 microns × 460.7 microns or a 40×: Zeiss Fluar 40× 1.30 N.A. Oil with a field of view of 230.3 microns × 230.3 microns. Settings (i.e., pinhole size, exposure time, gain, scan time, dwell time, etc.) were optimized for each sample in order to image the finest features of each sample (the neurite processes). Multiple z-stacks (5–20 μm plane-to-plane separation, depending on the objective used—20× images were 10–20 microns and 40× 5–10 microns) were acquired from the different culture conditions, and were exported as AVI files with 512 × 512 pixels (per z-slice/frame) and 24-bit color depth. For each sample, a 100 μm thickness was analyzed in a section away from the top or bottom surface of the sample in order to avoid edge effects. Confocal images were viewed using LSM 5 Image Browser (Zeiss).

### Segmentation test conditions

**Figure 1:**
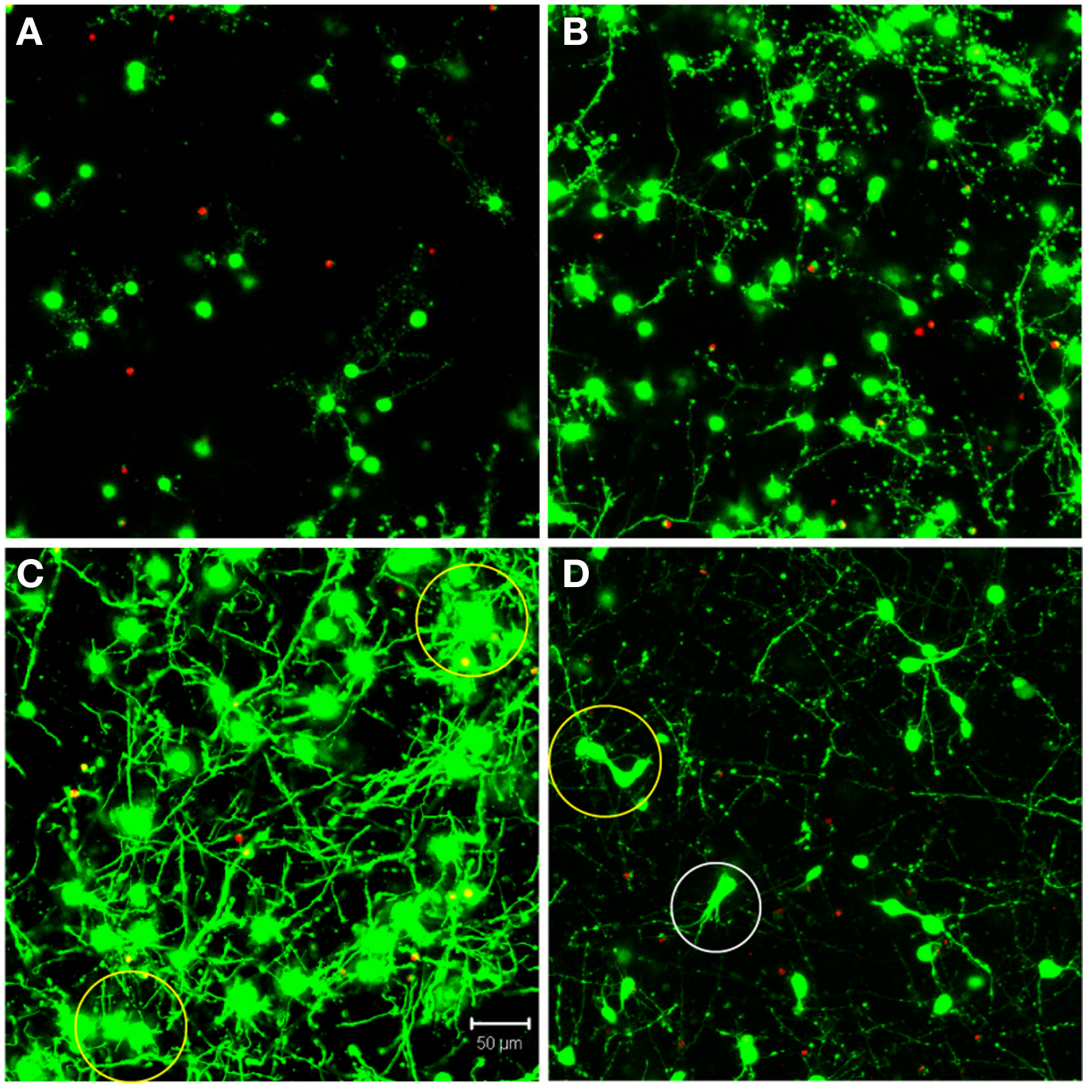
**Definition of test categories**. Variation in testing conditions was achieved by dividing cultures into four categories based on culture complexity. Levels one through three consisted of mainly spherical neurons with increasing amounts of neurite outgrowth. Specifically, **(A)** level one had spherical neurons with few neurites and no cell clustering; **(B)** level two had mainly spherical neurons with increased neurite outgrowth and little clustering; **(C)** level three had robust neurite outgrowth with increased cell clustering (yellow circles). Additionally, **(D)** level four had increased complexity due to more diverse, non-spherical neuronal morphologies (white circle), cell clustering (yellow circle), and significant neurite outgrowth. Images are 2D reconstructions of confocal z-stacks (100 μm total thickness) imaged at 20×; scale bar = 50 μm.

The automated segmentation routines were written in Matlab (Mathworks, version 7.01) and tested on 16 confocal z-stacks that were divided into four levels of culture complexity. Various complexity levels were tested to examine whether increasing complexity in neuronal morphology and neurite outgrowth (network formation) to examine the capabilities of our complex algorithms to minimize error (Figure 1). All levels utilized 3-D cultures of primary cortical neurons homogeneously distributed throughout thick (>500 μm) matrices. Different culture parameters such as matrix type (bioactive Matrigel/collagen-laminin and relatively bio-inert agarose) and cell seeding density (3750–5000 cells/mm^3^) resulted in morphologically different cultures. The variables leading to different culture levels were cell density/clustering (a function of matrix and cell seeding density; impacting degree of overlapping somata) neurite outgrowth (a function of matrix permissiveness; influencing process, non-cell soma, counts), and neuronal morphology (a function of matrix; altering shape of cell soma). The first three categories (of four) utilized agarose as the matrix material, and resulted in the maintenance of a spherical or near-spherical neuronal morphology. Level 4 cultures were developed within a bioactive Matrigel/collagen-laminin matrix where neurons were able to actively remodel and thus assume a variety of complex *in vivo*-like (e.g., non-spherical) morphologies not present in levels 1–3. The difference among levels 1, 2, and 3 are the amount of neurite outgrowth and resulting cell clustering. Specifically, level 1 represented a baseline with spherical neuronal morphology throughout culture, relatively low cell density (i.e., little clustering) while demonstrating a paucity of neurite outgrowth. Level 2 cultures had a moderate increase in neurite outgrowth with cell densities and morphologies similar to level 1. Level 3 cultures demonstrated extensive neurite outgrowth with an associated increase in cell density. Level 4 cultures also demonstrated significant neurite outgrowth, and although these cultures had a moderate cell density, there was cell clustering in some cases. Culture level descriptions are summarized in Table 1. In addition to testing multiple categories of *in vitro* cultures, we assessed the algorithm robustness by applying the automated segmentation routines to confocal z-stacks generated from *ex vivo* brain slices.

**Table 1:** **Description of 3-D neuronal culture parameters by category number**.

	**Soma morphology**	**Neurite outgrowth**	**Cell clustering**
Level 1	Spherical	Low	Low
Level 2	Spherical	Medium	Low
Level 3	Spherical	High	Medium
Level 4	Complex	High	Medium

*Higher category numbers correspond to increased culture complexity*.

### Validation methodology and statistical analysis

For validation of software performance, the total cell count, fraction of false positive cells, and fraction of false negative cells were recorded at various stages in the routine. The accuracy and error percentage were calculated in comparison to manual counts attained by experienced technicians. Validation was performed on all 16 culture confocal z-stacks as well as 2 brain slice z-stacks. We chose a conservative validation approach to analyze the total error present at various stages of the algorithm. Specifically, the error analysis was based on the total error percentage, defined as the percentage error based on the sum of the number of false positives plus the number of false negatives (calculating accuracy based purely on count output may artificially raise performance as false positives and false negatives can potentially cancel out). Two-Way repeated measures general linear model ANOVA was performed with culture complexity (i.e., level 1–4) as an independent variable, sub-routine point as the repeated variable, and count accuracy, false positive (%), false negative (%), and total error (%) as dependent variables. When significant differences existed between groups, Tukey's pair-wise comparisons were performed. For all statistical tests, *p* < 0.05 was required for significance. Data are presented as mean ± standard deviation.

### System development

**Figure 2:**
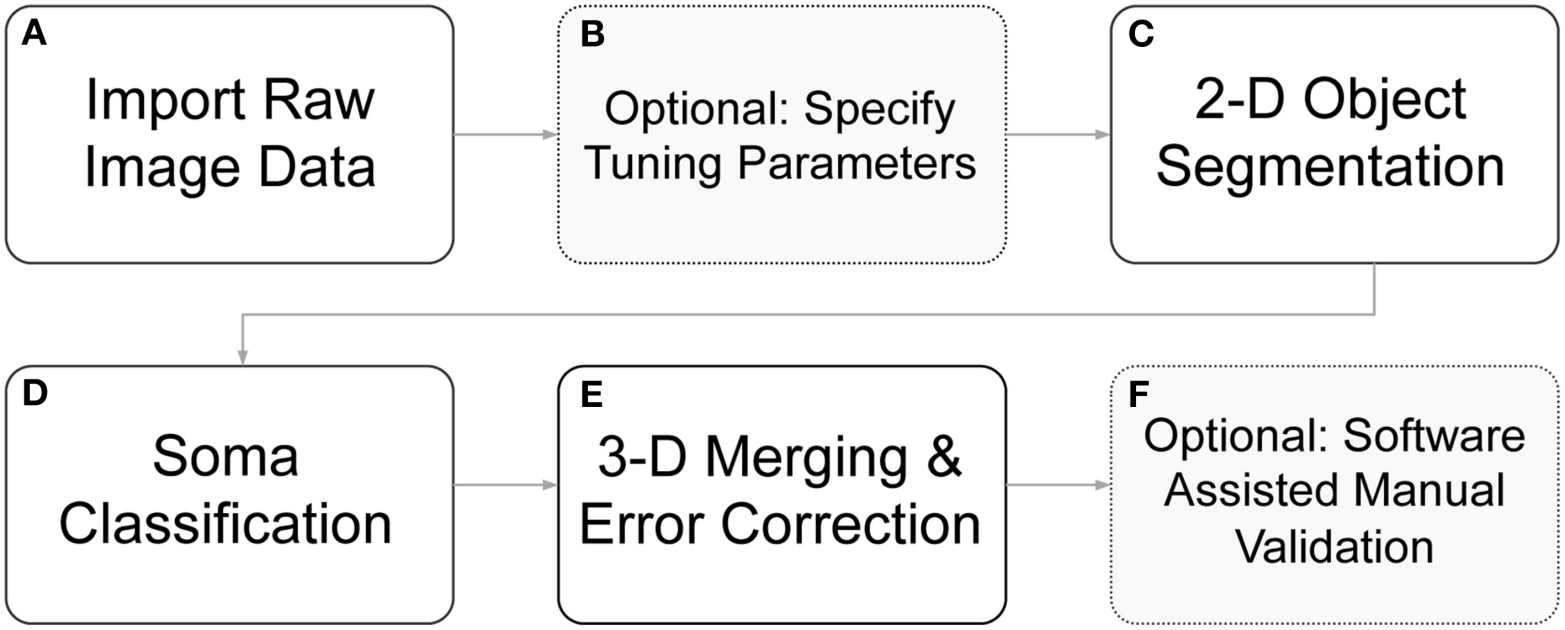
**Flow diagram of segmentation routines. (A)** Raw image z-stacks were imported into the program. **(B)** Prior to initiating cell segmentation, the user could optionally specify segmentation tuning parameters (otherwise default parameters were used). **(C)** Objects of interest, including potential somata, were identified in each 2-D frame in the z-stack. **(D)** The segmented objects were further separated into somata *candidates* and neurite fragments. **(E)** 2-D soma *candidates* were evaluated against objects in neighboring z-slice and were either stitched together into 3-D objects or removed from consideration. **(F)** Following automated segmentation and error correction, the user could choose to manually correct any remaining segmentation errors with click-commands from the mouse. During the 3-D merging routines, if an object failed to meet certain criteria, it was flagged to alert the user to a possible error.

We have developed algorithms to identify cell body boundaries for images possessing rich morphological detail and dense neurite outgrowth. For labeling 3-D cellular objects in a confocal stack of 2-D images (z-stack), we considered two different strategies: (1) segmenting cells in 2-D slices and merging overlapping cellular objects (Tekola et al., 1996; Irinopoulou et al., 1997; Belien et al., 2002), and (2) segmenting cells in 3-D volumes (Sarti et al., 2000; Adiga and Chaudhuri, 2001; Chawla et al., 2004). We choose the former because an analysis of this type easily lends itself to visual feedback and rapid error correction, and the merging of 2-D “blobs” into 3-D cellular objects provides an opportunity for error identification and correction. Furthermore, 2-D segmentation with 3-D “stitching” can be computationally much less expensive than 3-D segmentation and may enable more rapid processing. Undoubtedly, some of these advantages are not exclusive to this strategy, but they do more naturally fall out from this approach, which greatly simplifies algorithm implementation. User feedback and rapid, click-and-correct error correction were required for our applications, where both high throughput and near perfect accuracy are desirable. The ability to rapidly identify cell bodies, at any point during a live experiment, influenced this work significantly. An overview of the full segmentation process—including 2-D segmentation, 3-D merging, automated error correction, and software assisted error correction—is presented in Figure 2.

### 2-D somata segmentation and neurite filtering

Although the 3-D merging of 2-D cellular objects can compensate for errors in 2-D segmentation, it is desirable for 2-D cell body boundary identification to be as accurate as possible. For each of the test categories, accurate identification of cell boundaries required image preprocessing, 2-D watershed segmentation, and object classification and neurite filtering, which are described in detail below.

### Color filtering and global thresholding

**Figure 3:**
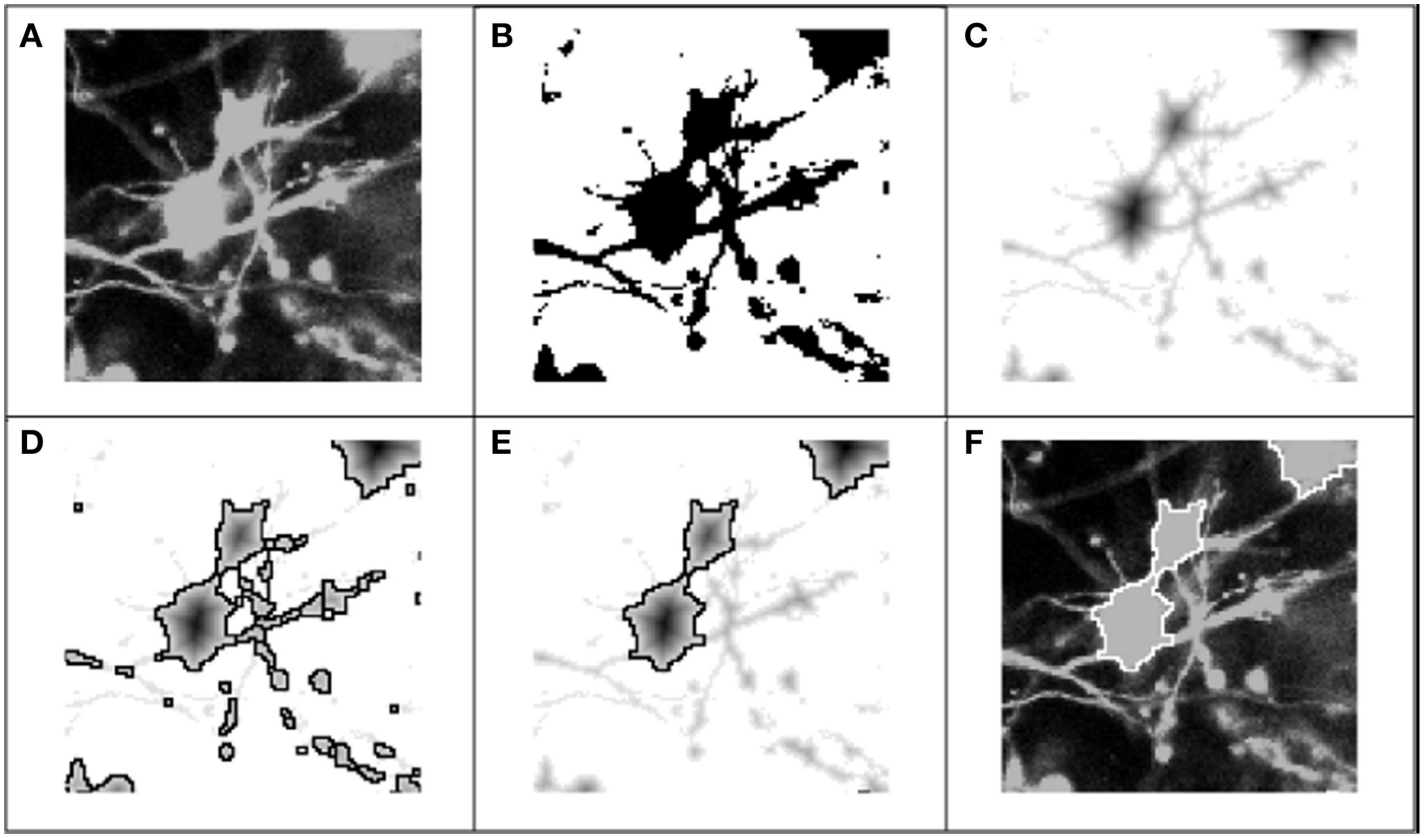
**Process flow for 2-D segmentation of cell bodies**. Graphic representation of 2-D segmentation process: **(A)** The color component for the fluorescent dye of interest was extracted to form an achromatic intensity image; **(B)** A global threshold was applied for each 2-D frame in the z-stack, separating pixels into foreground (regions of interest) and background; **(C)** The regional minima were defined by applying the Euclidean distance transform (or alternatively the Chebyshev transform) to the “thresholded” image; **(D)** Following application of the distance transform, the watershed algorithm was applied to the transformed image: the mottled contours of the neurites produced very fractured segmentation boundaries, while the rounder, smoother morphologies of the soma produced accurate segmentation boundaries; **(E)** Objects were classified as either soma or neurite fragments according the area enclosed by the watershed lines, and neurite fragments were removed from consideration. **(F)** Picture of the cell body boundaries projected back onto the original image.

As a first step toward identifying somata, we used color and intensity cues to separate the image into foreground and background regions. Through the graphical interface, the user scrolled through color-specific intensity histograms for each 2-D frame in the z-stack. For a specified dye, the color component was extracted, and each frame was transformed into an achromatic intensity image (Figure 3A), whereupon a global intensity-based threshold, T, was applied to separate the image pixels into foreground and background (Figure 3B). Pixels with an intensity value above the threshold were identified as potentially belonging to somata, and were assigned a value of 1. Pixels below the threshold were assigned a 0 or background value: I(x, y) = {1 I ≥ T; 0 Else}, where *x* and *y* represent the pixel indices within a 2-D image frame. Following the application of the threshold, the remaining objects in the binary image consisted of somata, neurites, and image artifacts.

### Optional morphological filtering

After separating images into foreground and background regions, morphological operators can be applied to remove holes inside remaining objects (and to separate overlapping objects that are connected with very narrow regions). In our test images, there was very little intensity variation among the pixels that represented somata; therefore, the application of a global threshold did not produce holes in the foreground objects. (In order to capture images with neurites and rich morphological detail, we used a high intensity light source during image acquisition, and, as a result, many of the soma and neurite pixels were at or near saturation.) Thus, we did not require morphological filtering to reshape the binary images; however, in the case of level 3 images, where cell clustering was rampant and neurite outgrowth pervasive, we used a 3 × 3 binary kernel with the center element and 4 nearest neighbors set to 1 (i.e., a “diamond” shaping-element) to erode the binary images. Successful usage of morphological filtering on level 3 cultures indicates that flexibility of the system to work independent of morphological filtering, culture morphology, or image saturation.

In general, there may be advantages to avoiding morphological filtering, as the resulting smoothing can remove spatial cues that naturally indicate overlapping objects (Crespo and Maoio, 1999; Kumar and Shunmugam, 2006). Given the lack of intensity variation within the soma of our images, morphological filtering was generally not needed. Without morphological filtering, “necklines” and other inflection points were preserved to help properly define the regional minima that guide object segmentation, but the morphological filtering performed in level 3 cultures showed that the algorithm is not reliant on preserving this information or oversaturated images. So, while the successive use of dilation and erosion operators may help remove some neurite features, such measures may also remove critical boundary indicators.

### Distance transforms

The successful application of the watershed algorithm requires that each object is marked by a regional minimum and that the image contours more or less follow the object boundaries. Unfortunately, natural intensity gradients are not sufficient to define the regional minima for each object (Lin et al., 2003). This was particularly true of our test images, where nearly saturated pixels presented very little texture in the soma region. Regardless, the analysis tool was built to function properly independent of a saturated or unsaturated soma region. To derive the regional minima and object contours, the Euclidean distance transform was applied to the stack of binary images (Figure 3C):

(1) $$\text{I}\left(x,y\right)=\sqrt{{\left(x-x_{b}\right)}^{2}\;+\;{\left(y-y_{b}\right)}^{2}}$$

where *x_b_* and *y_b_* represent the coordinates of the nearest background pixel. The local minima were assigned to pixels with the maximum distance value to the nearest background pixel. For most test images, the Euclidean transform produced satisfactory results. However, for images with a high degree of clustering and dense neurite outgrowth (level 3 and level 4), the Chebyshev or “chessboard” transform was used to minimize over-segmentation errors:

(2) $$I(x,y)=\text{max}\left(\left|x-x_{b}|,\;|y-y_{b}\right|\right)$$

The Euclidean distance transform accounts for the projection along both the x- *and* y-axes between a given pixel and its geodesic distance to background. Because the Chebyshev transform defines values based on the maximum projection along the x- *or* y-axis, the determination of the local minima may be more immune to erratic variations along one axis. Thus, images with extremely rich and complex morphologies appear to be less susceptible to segmentation errors when the Chebyshev transform is applied.

### 2-D watershed segmentation and object classification

After the regional minima were defined by the transformed images, we applied a 2-D watershed algorithm to segment the objects (Figure 3D). The watershed algorithm involves interpreting the image as a surface in which points of the same intensity value are at the same height, and classifying points according to the direction of the gradient at their respective locations: (1) regional minimum, points that reside at a local minimum of the surface, (2) “catchment basins,” points whose gradients point in the direction of the same minimum, and (3) “watershed lines,” points that reside at a local maximum, and thus could belong to any adjacent minima. A common analogy for describing the watershed algorithm involves punching a hole in each regional minimum and then flooding the entire image from the bottom. Watershed lines or segmentation boundaries are then constructed to prevent distinct flooding regions from overlapping. Many details on 2-D and 3-D watershed implementations have been previously described (Adiga and Chaudhuri, 2001; Gonzalez and Woods, 2002).

**Figure 4:**
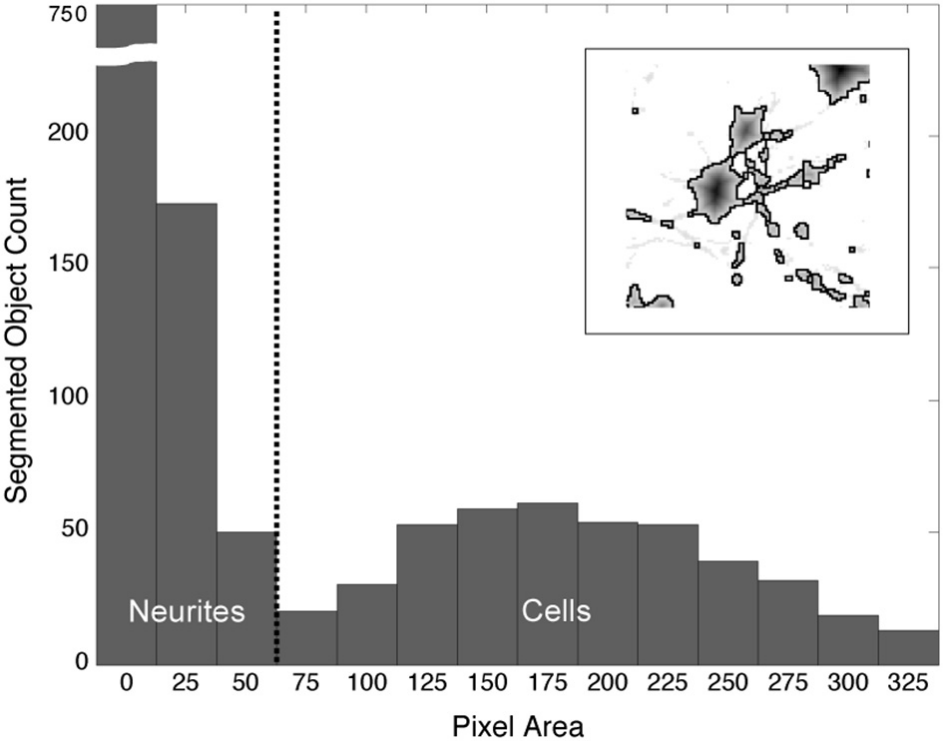
**Distribution of segmented objects binned according to pixel area**. The morphological differences between neurites and somata produce watershed segmentation boundaries that, when binned according to pixel area, fall into two distinct populations. The threshold, α (vertical, dashed line), is used to separate objects into neurites and cell bodies. Objects with a pixel count or area ≥ α are labeled “cells”; objects with an area < α are labeled “neurite fragments.” (Inset) Watershed boundaries for somata and neurite fragments.

The watershed algorithm produced segmentation boundaries that were related to the image contours. Whereas, the mottled contours of the neurites resulted in highly fractured objects, the relatively smooth morphologies of cells produced larger and rounder objects. It was on the basis of these differences that we easily distinguished between object types. Figure 4 demonstrates a typical bi-modal distribution for segmented objects binned by pixel area that is presented to the user. Based on the histogram, the user can intelligently set the area threshold based on their particular microscope, culture, or tissue conditions. Objects less than the area threshold, α, were labeled neurite fragments, and objects greater than α were labeled cellular objects. Subsequently, all segmentation boundaries corresponding to objects with areas lower than α were dismissed from consideration (Figure 3E), and the result was visualized superimposed on the original image (Figure 3F). The populations were not perfectly distinct, so there was a small probability that some neurite fragments remained; likewise, a few small cell bodies could have been removed. We remedied the inclusion of segmentation errors by observing idiosyncrasies that arose when merging 2-D objects into 3-D cell bodies.

### 3-D merging and error identification

**Figure 5:**
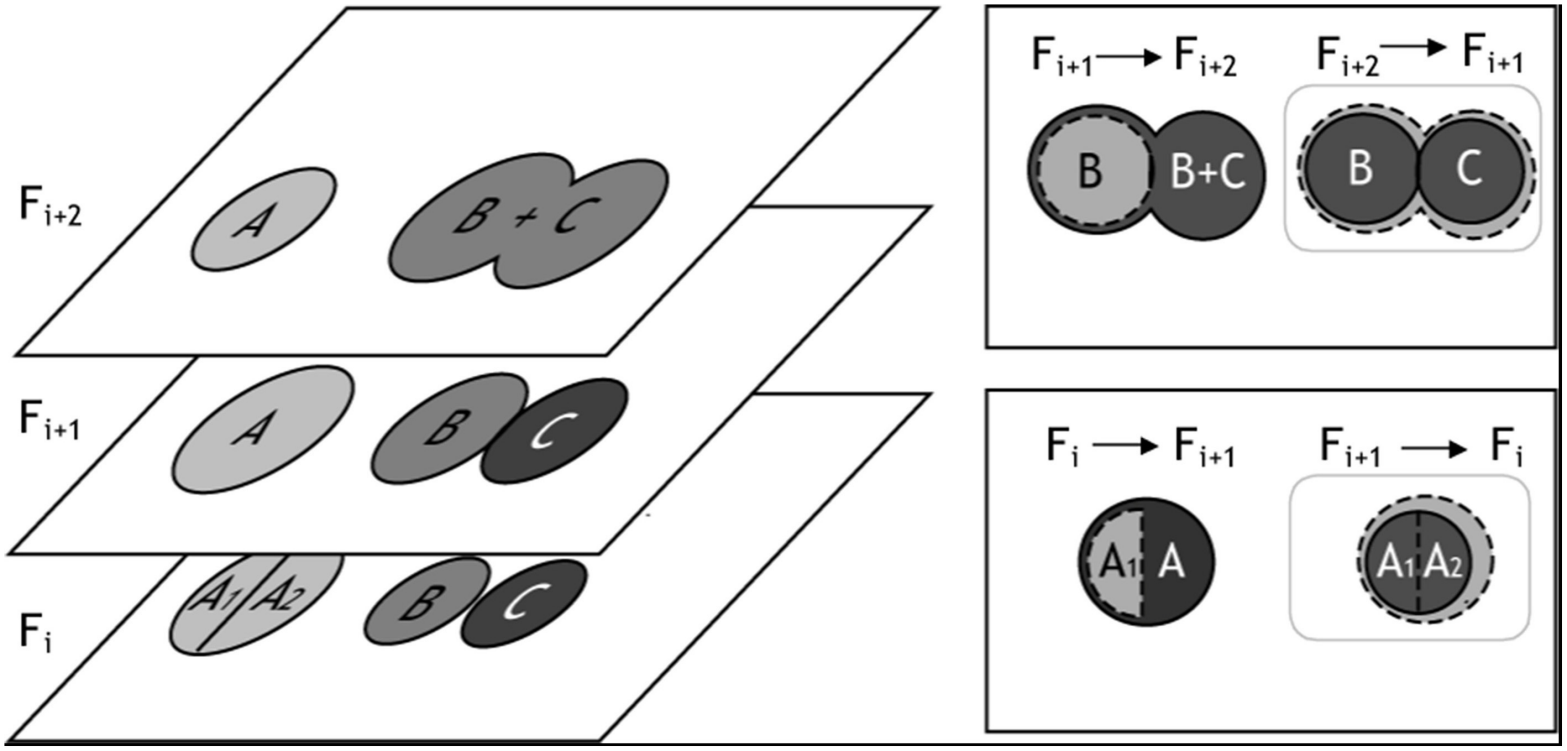
**Illustration of 3-D merging and error identification. (Left)** This figure exemplifies segmentation results for three cells—A, B, and C—which appear in z-slices/frames Fi, Fi+1, and Fi+2. In Frame Fi+1, we show three correctly segmented somata. In Frames Fi and Frames Fi+2 we illustrate over-and under-segmentation errors, respectively. 2-D objects are merged into 3-D cell bodies if the percentage overlap between the objects is ≥ β, where β can be any number between 0 (no overlap) and 1 (100% overlap). The merging algorithm considers two z-slices/frames at a time, and segmentation errors are identified when multiple objects in a single frame exceed β. (**Top Right**) For example, cell B from frame Fi+1 was projected into Fi+2. All object(s) in Fi+2 that overlapped with B's projection (dashed cell boundary) were considered as merging candidates; in this case, the object B + C satisfied the percentage overlap criteria. However, the reverse projection of B's best merging candidate, B + C, back onto the previous z-slice/frame identified two objects that satisfied the merging criteria: B and C. Because three objects - B, C, and B + C - were eligible for merging, the under-segmentation error was identified (gray box). (**Bottom Right**) In a similar fashion, the forward projection of A1 into Fi+1 overlapped best with A; however, the reverse projection of A1's best merging candidate significantly overlapped with two objects: A1 and A2, thus identifying a 2-D segmentation error.

Following watershed segmentation and object classification, each 2-D frame contained segmented objects that fell into one of three categories: (1) correctly segmented somata, (2) false positives (neurites, artifacts, and over-segmented cells), and (3) false negatives (under-segmented somata and unidentified cell bodies). To finalize the segmentation of cells in three dimensions, we merged 2-D objects into 3-D cell bodies, and we used conflicts that arose during merging to identify and, in most cases, automatically correct segmentation errors (Figure 5). The algorithm for merging 2-D “blobs” into 3-D cells involved assessing the merger from two vantage points: (1) the *forward projection* of a 2-D cellular object onto overlapping objects in the adjacent frame and (2) the *reverse projection* of overlapping objects back into the original frame. These two vantage points ensured that only cells with maximum *mutual* overlap were merged. (A scenario, which we refer to as “unrequited overlap,” can arise where an object, C_1_, in Frame F_i_ maximally overlaps with another object, C_2_, in Frame F_i+1_ which shares the greatest maximum, mutual overlap with yet another object, C_3_, in Frame F_i_).

In addition to the mutuality criteria for merging 2-D objects into 3-D cell bodies, we required that the percentage of object overlap between 2-D objects exceed an empirically defined threshold, β —defined as the proportion of the intersecting pixels, Area(c_i_ n c_i+1_), to an object's pixel count, Area (c_i_). If *multiple* objects from either vantage point exceeded β, then a segmentation error likely occurred. Any number of actions could then be taken to resolve the conflict, such as merging over-segmented objects, splitting under-segmented objects, or flagging the offending object pixels for manual user validation (at the conclusion of all automated routines). For our application, we choose a simple mode of action: for all errors identified in the forward projection, we merged objects *and* set flags for user validation; for errors identified in the reverse projection, we only flagged the offending objects for user validation. (Empirically, it was determined that most identified errors required merging but that under-segmentation, which requires splitting, was more likely to be identified in the reverse projection.) The threshold, β, took on any value between 0 (no overlap) and 1 (complete overlap), where the smaller the parameter implemented, the more sensitive the algorithm was to potential errors. In any frame, if a 2-D cellular object was not connected to pixels in the adjacent frame(s) it was assumed to be an artifact or neurite and was removed. The algorithm is summarized in pseudo-code form below:


Procedure 3Dmerge_ErrorCheck
Fi = Frame in z-stack
  C_Fi_ = {Set of 2-D objects in Frame, Fi}
  C^j^_Fi_ = 2-D object in C_Fi_
Initialize: E_Fi_ = {0}; Set of
                         objects marked for error
Initialize: Ce_Fi_ = C_Fi_ ∀ Fi; Set of objects
                       eligible for 3-D merging
For each Frame in the z-stack, Fi
  For each *eligible* cell, Ce^j^_Fi_, in the current
  Frame, Fi
    Fk = Fi
    Repeat
       1. Project Ce^j^_Fk_ onto F_k+1_
          Co←Ce^j^_Fk_ ∩ C_Fk+1_; Co is the set of
                      overlapping objects in C_Fk+1_
             If objects in Co exceeding β, Cb, are
                         greater in number than 1
               A. Merge Cb in 2-D
               B. Flag objects for error check;
                  E_Fk+1_ ← E_*Fk*+1_ ∪ Cb ∪ Ce^j^_Fk_
       2. Project Cb onto F*_k_*
          Co ← Cb ∩ C_Fk_; Where Co is set of
                      overlapping objects in C_Fk_
             If objects in Co exceeding β, Cr, are
                         greater in number than 1
               A. Flag objects for error check;
                                     E_Fk_ ← E_*Fk*_ ∪ Cr
       3. If Ce^j^_Fk_ AND Cb share the greatest
                                    mutual overlap
               A. Merge Ce^j^_Fk_ and Cb in 3-D
               B. Remove Cb from the eligibility
                                    set, Ce_Fk+1_
               C. Increment Fk
Until Ce^j^_Fk_ is NOT attached to a new object in
                                  3-D (Step 3A)


### User interface and software assisted error corrections

**Figure 6:**
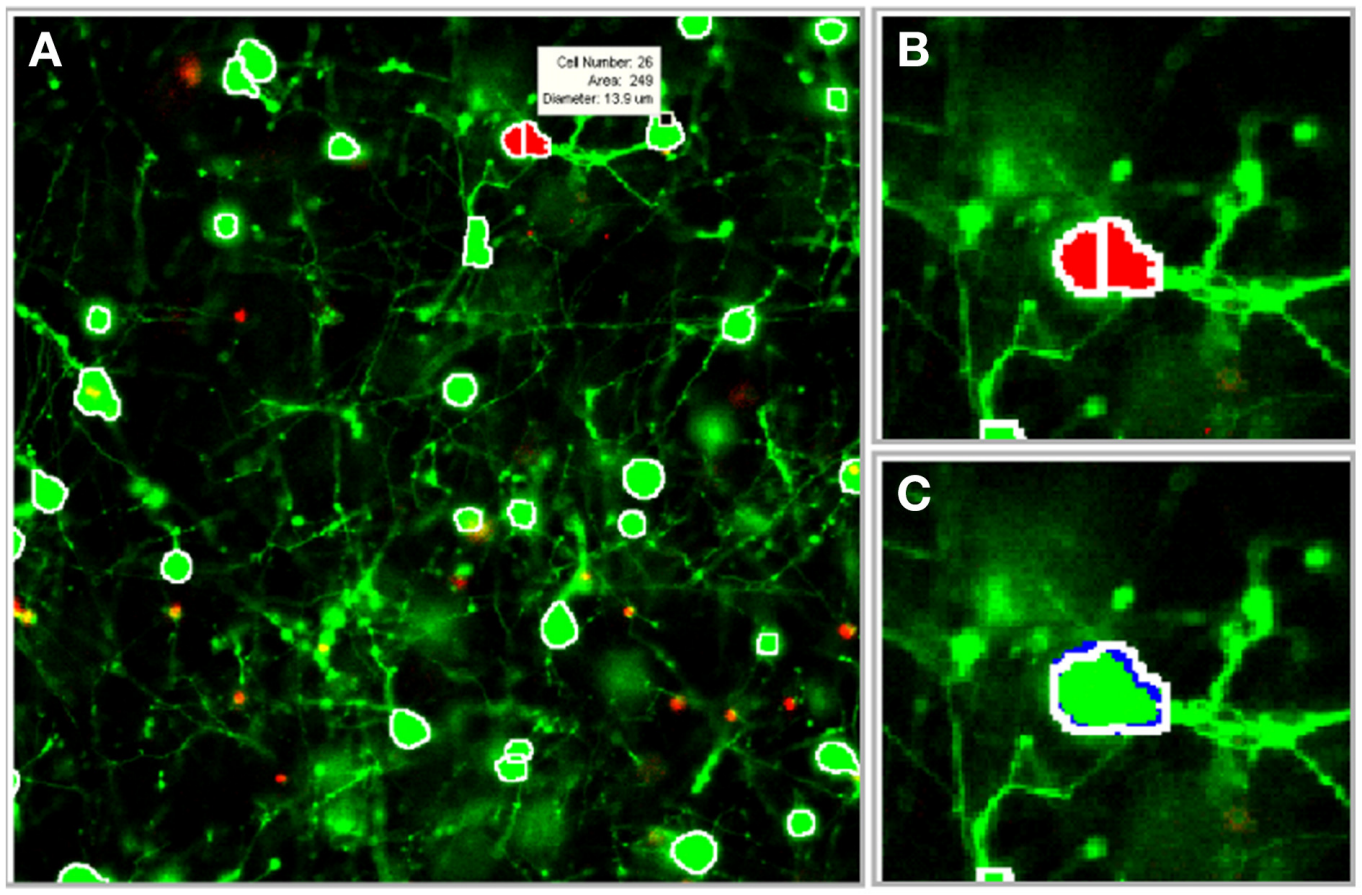
**Illustration of software tools**. The segmentation algorithms were integrated into a graphical user interface (GUI) to facilitate visual feedback, parameter selection, and software assisted error correction. **(A)** Segmentation boundaries (white) are projected onto individual frames in the z-stack (a scroll bar, not shown, is used to switch between frames). Segmentation data, including cell ID number, area, and diameter (white box), are displayed for a selected cell. **(B)** Potential segmentation errors are automatically flagged in red. **(C)** A user-applied mouse command instructs the software to perform a merge operation. Blue pixels outline the object in the forward adjacent z-slice/frame that is connected to the merged cell. (Gray pixels, not shown, indicate connected objects in the previous frame). Images were taken at 20×.

The image processing algorithms discussed previously were integrated into a graphical user interface (GUI) that was designed to facilitate parameter selection, visual feedback, and user-guided edits (Figure 6). The following features were incorporated into the GUI: (1) 2-D and 3-D cell databases for maintaining cell coordinates and boundary information, (2) histograms and segmentation statistics for assisting parameter selection, (3) saving and reloading options for revisiting and revising z-stacks, (4) computer assisted manual segmentation for error correction, (5) morphological operator and process selection for preprocessing, and (6) 3-D idealized graphic reconstructions of segmented cultures. Although it is desirable for the software to achieve 100% accuracy with zero user intervention, software guided edits enable rapid adjustments or corrections to the segmentation parameters and results. (*The program is available upon request to the authors*).

### Software assisted error corrections

During the 3-D merging routine, a database was constructed to catalog information about each cell, including its geometric properties (such as eccentricity and concavity) and pixel coordinates. The benefits of the database were twofold: (1) it provided valuable statistics and information about the segmented cells, and (2) it was useful for rapid visual feedback and user-guided corrections. The indices that corresponded to the boundaries of segmented objects were projected onto the original 2-D images in the z-stack. The user validated the images by scrolling through each 2-D z-slice/frame to observe the segmentation boundaries. To expedite manual edits, conflicts identified during 3-D merging were flagged (with red pixels) to draw attention to the most probable areas that required user input. The mouse was used to display statistics about a suspicious or flagged cell (such as cell ID number, area, equivalent diameter) to help assess the accuracy of a particular cell boundary. Following a decision about the accuracy of the automated boundary, the following mouse commands were used to manually edit the image:


Left click: add/delete cell in current 2-D frame
Middle click: merge over-segmented 2-D cells
Right click (and hold): manually draw cell boundary


Each correction, addition, or deletion of a cell in a *2-D* z-slice/frame evoked a cascade of procedures that managed the creation, deletion, merging, and splitting of *3-D* cells. Cells were automatically merged in 3-D if they satisfied the mutual overlap criteria (as defined previously). Segmentation lines for 2-D cells in adjacent z-slices that were merged to a user-selected cell were color-coded and displayed in the current z-slice. These methods allowed the user to confidently produce near 100% accuracy in very little time. This is especially useful in applications where highly accurate on-line cell segmentation and identification is required for optical tracking of network activity.

### Software assisted manual segmentation

The same set of software tools that enabled automated segmentation with click-and-correct manual edits were used for an altogether different method of cell segmentation: software-assisted *manual* segmentation. For this application, the 2-D automated segmentation routines were executed (without 3-D merging routines) to record the indices of *potential* cells. In order to prevent biasing the user, visual feedback was suppressed since the outline of cells was not shown, and the user clicked on individual cells in each 2-D frame to indicate which objects were cells. The index of the user's click was compared against a 2-D database of potential cells; if a match was found, the automated segmentation boundaries of the potential cell were displayed. Click commands selected or de-selected the cell, provided an alternative (non-watershed based) segmentation boundary, or allowed the user to manually segment the cell. As the user moved between z-slices/frames, 3-D merge and split operations were automatically performed, and visual feedback was provided to indicate the relationships of cells between frames. This method of software-assisted segmentation allowed us to manually build a collection of test images with which to evaluate our automated routines. For each image evaluated, skilled technicians carefully identified each cellular object in both 2-D and 3-D.

## Results

The segmentation routines were rigorously tested in order to assess both the accuracy and robustness of the algorithms. Accuracy measures were attained by evaluating the efficacy of the algorithms both with and without error correction routines. Additionally, the influence of user-defined biases and the applicability of these algorithms to *ex vivo* brain slices were evaluated.

### Quantification of *in vitro* performance

**Figure 7:**
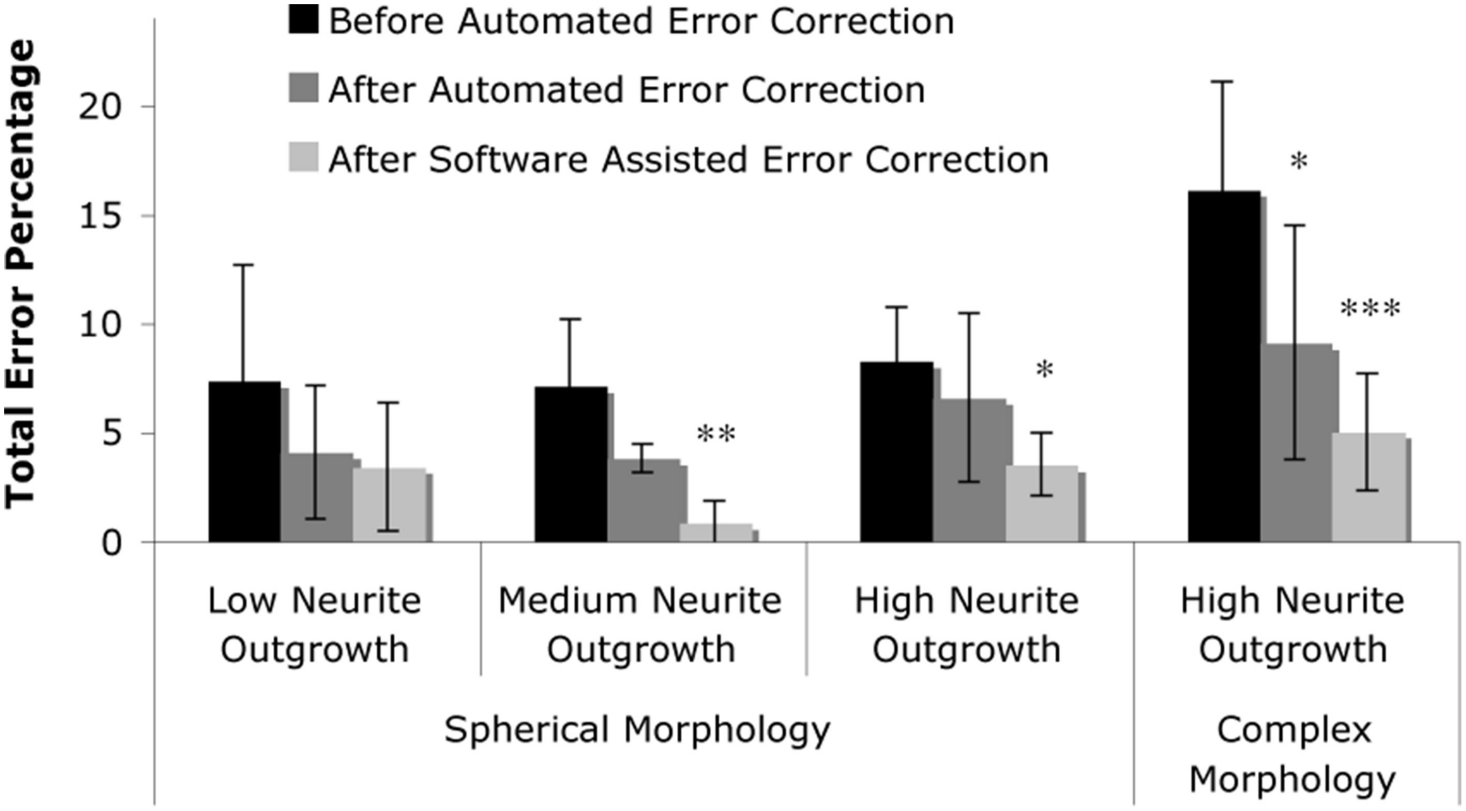
**Summary of results**. The total error percentage, defined as the number of false positives counts plus the number of false negative counts in comparison to the actual number of cells, was calculated for levels one through three (spherical morphology with increasing levels of neurite outgrowth) and level four (complex morphology with high neurite outgrowth). Two-Way repeated measures ANOVA revealed that the total error was reduced by the presence of automated error correction (*p* < 0.001), and was further reduced by correction of software-identified probable error points (*p* < 0.05). Tukey's *post-hoc* pair-wise comparisons revealed significant error reduction within levels two through four; asterisks denote significant reduction in total error percentage vs. “Before Automated Error Correction” within each level (^*^p < 0.05; ^**^p < 0.01; ^***^p < 0.001).

In order to assess the fidelity of the algorithms, automated software segmentation boundaries were compared to the software-assisted manual segmentation. Specifically, the results of the automated routines were evaluated without 3-D error correction, with 3-D error correction, and following manual correction of software-defined probable errors (manual correction was only allowed if the software flagged a potential error; in other words, errors that were not flagged by the software were ignored). At each of these stages, the following parameters were attained: (1) cell count, (2) over-counted cells (i.e., number of false positive cells), and (3) missed cells (i.e., number of false negative cells). Software defined probable error points were then manually assessed by experienced technicians and the appropriate action was taken (i.e., correction or no correction—although the vast majority of flagged errors required correction). The software quantification parameters (e.g., area threshold, α, and percent 3-D overlap, β) were empirically optimized for only one image-stack in each level, thus the same settings were maintained within each level to demonstrate robustness of the system (e.g., the output was consistent across samples for a give set of parameters). This multi-level analysis permitted assessment of the value of somata vs. neurite segmentation, 3-D context segmentation correction, and software-assisted user validation in significantly reducing error and enhancing the accuracy of the routine output (Figure 7 and Table 2).

**Table 2:** **Results**.

**Level**	**User defined parameters**	**Sample number**	**Actual number of cells**	**Cell count before automated error correction**			**Cell count after automated error correction**			**Cell count after correction of software identified probable errors**		
				**Count**	**False+**	**False−**	**Count**	**False+**	**False−**	**Count**	**False+**	**False−**
1	α = 60	01	26	27	2	1	25	0	1	25	0	1
	β = 70%	02	29	29	0	0	29	0	0	29	0	0
	T = 95%	03	28	26	0	2	26	0	2	26	0	2
		04	37	39	3	1	37	1	1	36	0	1
				**Mean error (%) =**	**4.0**	**3.4**	**Mean error (%) =**	**0.7**	**3.4**	**Mean error (%) =**	**0.0**	**3.4**
				**Stand.dev. (%) =**	**4.6**	**3.0**	**Stand.dev. (%) =**	**1.4**	**3.0**	**Stand.dev. (%) =**	**0.0**	**3.0**
2	α = 40	01	42	42	0	0	42	0	0	42	0	0
	β = 70%	02	66	68	2	0	68	2	0	66	0	0
	T = 95%	03	65	71	6	0	68	3	0	65	0	0
		04	52	54	4	1	51	1	1	51	0	1
		05	135	144	9	0	140	5	0	137	2	0
				**Mean error (%) =**	**5.3**	**0.4**	**Mean error (%) =**	**2.7**	**0.4**	**Mean error (%) =**	**0.3**	**0.4**
				**Stand.dev. (%)=**	**3.8**	**0.9**	**Stand.dev. (%) =**	**1.8**	**0.9**	**Stand.dev. (%) =**	**0.7**	**0.9**
3	α = 30	01	36	39	3	0	37	1	0	37	1	0
	β = 70%	02	54	50	0	4	50	0	4	51	0	3
	T = 95%	03	86	96	10	0	96	10	0	88	2	0
		04	87	86	2	3	85	1	3	86	1	2
				**Mean error (%) =**	**5.6**	**2.7**	**Mean error (%) =**	**3.9**	**2.7**	**Mean error (%) =**	**1.6**	**2.0**
				**Stand.dev. (%) =**	**5.4**	**3.5**	**Stand.dev. (%) =**	**5.3**	**3.5**	**Stand.dev. (%) =**	**1.2**	**2.6**
4	α = 15	01	36	39	5	2	35	1	2	34	0	2
	β = 35%	02	27	28	3	2	27	2	2	25	0	2
	T = 95%	03	48	49	3	2	46	0	2	47	0	1
				**Mean error (%) =**	**10.4**	**5.7**	**Mean error (%) =**	**3.4**	**5.7**	**Mean error (%) =**	**0.0**	**5.0**
				**Stand.dev. (%) =**	**3.9**	**1.6**	**Stand.dev. (%) =**	**3.7**	**1.6**	**Stand.dev. (%) =**	**0.0**	**2.7**

*For each image (sorted by test level), the number of false positives and false negatives were tabulated for three different conditions: (1) when no error correction was applied, (2) when automated error correction was applied, and (3) when both automated and software assisted error correction was applied*.

Fully automated and user-corrected quantification were compared to manually-attained cell counts in order to assess the overall accuracy and error sources of the software. The results are tabulated in Table 2, which depicts the raw counts for the different points of the sub-routine in addition to the false positive and false negative counts (including error percentages). Analysis of the total error percentage (based on false positive cells plus false negative cells) was found to be a sensitive measure of software performance. The total error percentage was found to depend significantly on cell culture level (*p* < 0.05) and sub-routine point (*p* < 0.001), with no interaction between these factors. Overall, the application of 3-D error correction significantly reduced the total error percentage (*p* < 0.001 for each). There was an additional significant reduction in the total error percentage when software-assisted error correction was applied following automated 3-D error correction (*p* < 0.05). Pair-wise comparisons within the four cell culture levels clearly demonstrated the importance of 3-D segmentation and error correction as culture complexity increases (Figure 7). However, such analysis techniques did not significantly improve performance in relatively simple samples (level 1) where somata assume spherical morphologies and bear few or no processes.

### Sensitivity to user-defined parameters

To investigate the influence of parameter selection on the accuracy of the segmentation routines, we examined cell count error as a function of the area classification criteria, α, and intensity threshold, T, for a level 2 test image. The area threshold was normalized to one standard deviation less than the mean of the cell bodies [i.e., 100% = Mean(cell bodies) – StdDev(cell bodies)], and the parameter was swept from 15 to 100%. The intensity threshold was swept from 35 to 100% of the maximum pixel intensity. Error was defined as the percent deviation of the automated cell count from the “gold standard” cell count, and was tabulated for the segmentation algorithms run both with and without automated 3-D error correction.

**Figure 8:**
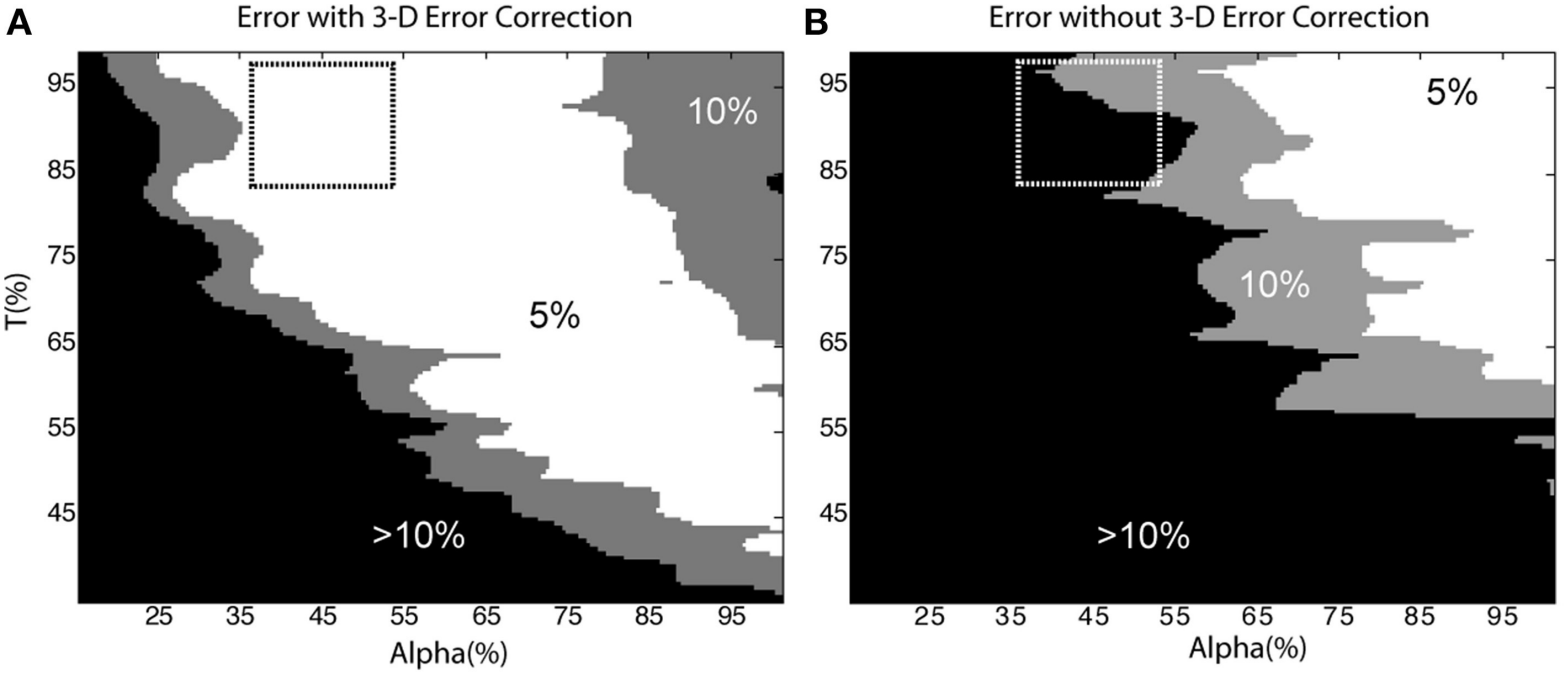
**Sensitivity to user-defined parameters**. Cell count error as a function of α and T with **(A)** and without **(B)** 3-D error correction. The dashed box indicates the parameters an operator would likely select based on histogram data from the software. In **(A)** the 5% error region occupies 44% of the shown parameter space; in contrast, the same error region occupies only 18% of **(B)**. The removal of neurite segments and the merging of over-segmented somata in **(A)** accounts for the reduced sensitivity to user defined parameters.

Figure 8 depicts error as a function of the user-defined parameters, α and T (the dashed-box indicates parameters that were most likely to be manually selected based on histogram feedback provided by the software, for example, refer to Figure 4). In general, the automated cell count increased as the thresholds were decreased. Thus, in the case without 3-D error correction, the cell count was artificially raised by a lower threshold and the inclusion of over-segmented somata and neurite fragments, which was corrected for in the case with 3-D error correction. Figure 8 demonstrates that using the 3-D context to identify and correct segmentation errors generates a much more robust and resistant parameter space with which to achieve accurate cell-count results. Therefore, there is a larger error tolerance for parameter selection, and future versions of the software may be able to automate parameter selection.

### Performance validation in brain slices

**Figure 9:**
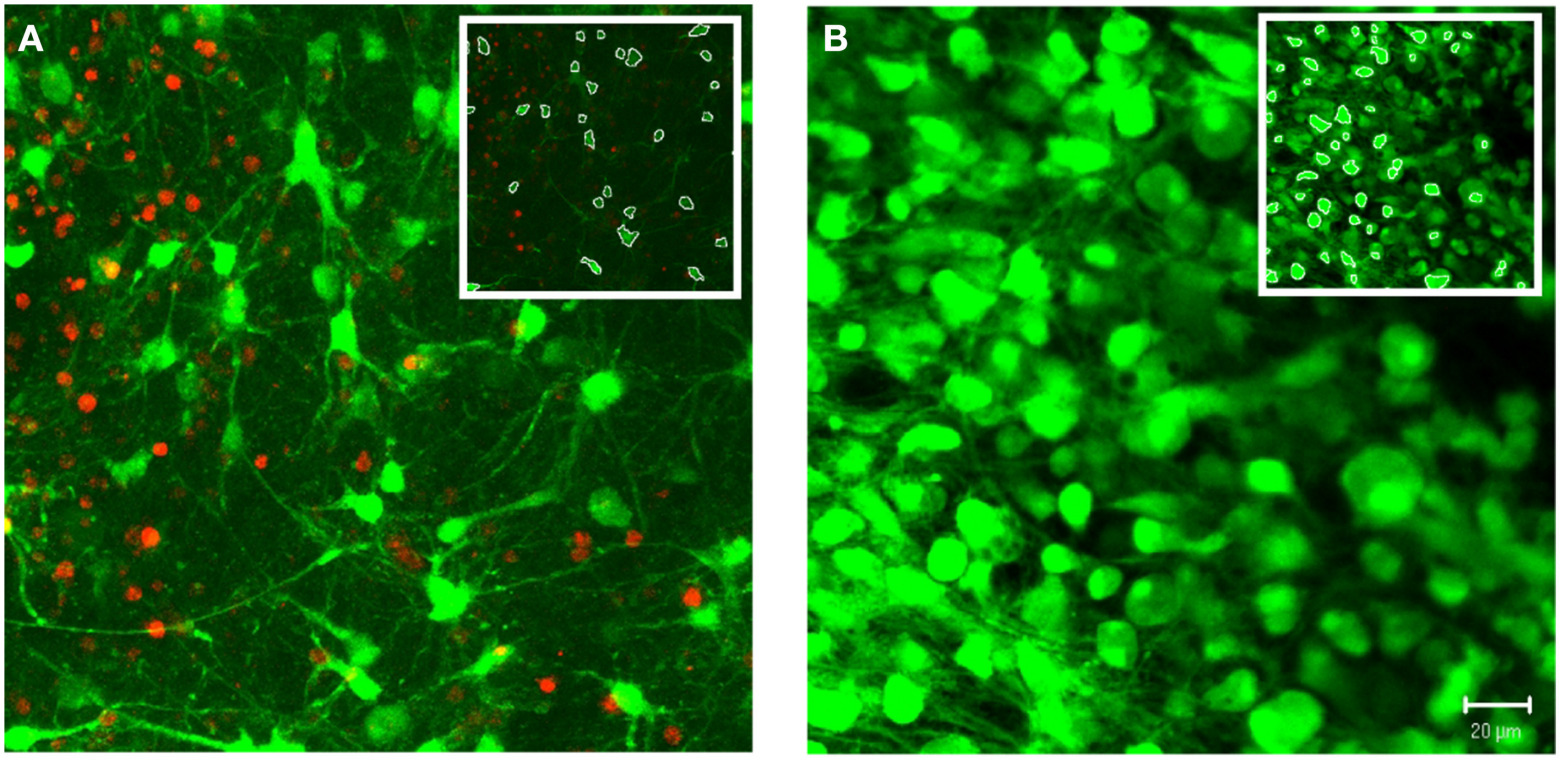
**Demonstration of system robustness: soma segmentation in** ***ex vivo*** **brain tissue**. Custom 3-D segmentation algorithms were applied to z-stacks attained from confocal imaging of brain slices from cerebral cortex. The z-stacks tested varied based on the density of viable cells, ranging from relatively low **(A)** to high **(B)** densities. Following the same protocol of the *in vitro* testing, the algorithms achieved accuracies of 93% **(A)** and 97% **(B)** (α = 50, β = 0.15, T_A_ = 60%, T_B_ = 95%). Images were taken at 40×. (Inset) White pixels indicate soma boundaries for a single slice within the z-stack. Scale bar = 20 μm.

In addition to 3-D *in vitro* cultures, the image processing algorithms were also applied to 3-D *ex vivo* brain slices. As a demonstration of system robustness, the algorithms were tested on confocal z-stacks acquired from cerebral cortical brain slice cultures. We choose samples with varied densities of viable cells, ranging from relatively low to high densities (Figure 9). The z-stacks tested also varied based on range of morphologies and the contrast in cell features (e.g., cell pixels ranged from relatively faint, low intensity, to saturated, high intensity). Apart from the global intensity threshold, T, the user-defined parameters were identical for these two image stacks (α = 50, β = 0.15, T_A_ = 60%, T_B_ = 95%). Following the same protocols defined for the *in vitro* cultures, the software achieved 93 and 97% accuracies for the low and high cell density z-stacks, respectively.

## Discussion

We have demonstrated novel methodologies for the systematic and unbiased identification of neural cells distributed throughout thick 3-D tissue or *in vitro* constructs. We utilized novel features, including neurite/soma classification, 3-D merging and segmentation error identification, and software assisted user validation, to attain highly accurate cell boundary identification over a wide range of morphological culture complexities. This toolset addresses a gap between automated segmentation routines for multi-cell *nuclear* images (with no neural processes present) and *single-cell* (or low density) images with rich morphological detail. Additionally, this toolset allows users to make rapid, computer-assisted manual corrections to the automated segmentation database, which is particularly attractive when highly accurate assessments of cell body locations must be made during a live experiment (e.g., optical tracking of network activity using Ca^2+^-sensitive dyes). We further validated these tools using the complex application of neural cell quantification in *ex vivo* brain slices. Moreover, we demonstrated that our algorithms leverage the increased complexity inherent in 3-D systems as an effective means to minimize quantification errors by applying the rich set of spatial data to automatically correct segmentation errors. Collectively, these techniques improve the scope and efficiency of automated neural-specific analyses without sacrificing accuracy by identifying neuronal somata across 3-D tissue or tissue surrogates in z-stacks preserving morphological/connectivity data. This novel 3-D image analysis platform offers neural engineers and neurobiologists a valuable set of tools for the analysis of neural tissue or tissue surrogates within a 3-D context, appropriate for the study of cell-cell and cell-extracellular matrix interactions.

While simple morphologies similar to somatic or nuclear staining did not require the novel error correction methods, the significant improvements in accuracy for complex neurobiological examples underscore the benefit of this toolset. Although other options exist, they lack capabilities of this toolset, namely the ability to examine complex neurobiological examples with somata and neurites with one fluorescent marker (Bjornsson et al., 2008; Luisi et al., 2011; Latorre et al., 2013). We have validated these tools in the specific application of automated segmentation of neuronal somata with dense neurite arborization within 3-D constructs. Despite a conservative validation scheme, the algorithms performed very well for a variety of test images, with an accuracy ≥ 95% over a wide range of morphological culture complexities. Similarly, in brain slices, we performed a proof-of-concept test of the algorithms with an accuracy > 90%. It is surprising that the lower density slice had a lower accuracy, but the image may have had a fainter signal for some cells and large-caliper neurites that could have contributed to the slightly reduced accuracy. Since we only ran the algorithm on two *ex vivo* samples, we cannot determine if the algorithm performs better on higher density slices. During the validation process, users were not allowed to correct unmarked errors that were otherwise obvious to the investigator. Thus, the accuracy of the presented algorithms should approach 100% in practical applications.

We have addressed the challenge of parameter selection by taking measures to ensure that the process parameters were relatively easy to tune. The empirically derived operator variables that influenced the performance of the segmentation routines included (1) the pixel intensity threshold, T, (2) the soma/neurite area threshold, α, and (3) the 3-D merging overlap percentage, β. The area and intensity parameters, α and T respectively, were assigned based on histograms generated by the software. The overlap percentage, β, was used primarily to specify the sensitivity of the software to segmentation errors. Although careful tuning of the parameters certainly improved performance, the accuracy of the software was relatively robust to changes in these parameters (as indicated in Figure 8). Thus, it was only necessary to tune the parameters for one z-stack in each test category.

One of the challenges in image segmentation is to define a metric for object classification. In order to optimize performance (speed and accuracy), we chose to distinguish between neurite fragments and somata using very simple criteria—object pixel area. Although area thresholds have been reported in the past to separate *nuclei* from *artifacts* (Adiga and Chaudhuri, 2000), we have shown that watershed segmentation produces boundary areas with remarkably distinct populations for *neurites* and *somata*. Despite the efficacy of this method, inevitably some cell body objects were excluded while some neurite fragments were included. The 3-D merging algorithms all but eliminated this problem. For example, objects which only appeared in a single z-slice were assumed to be artifacts or neurite fragments and were removed. Furthermore, the exclusion of cell bodies (false negatives) usually occurred near the cells termination in the z-axis where the cell possessed a very small 2-D diameter; in this case, the cell was usually represented in neighboring z-slices, where confocal slices captured a larger 2-D perspective of the cell (a typical cell occupied from 3 to 6 z-slices in our application, although this will be a function of confocal microscopy parameters).

We have developed novel techniques for merging 2-D objects into 3-D *somata* and identifying 2-D segmentation errors. These techniques improve on previous methods (Irinopoulou et al., 1997; Belien et al., 2002) to merge 2-D objects into 3-D *nuclei*. Specifically, we introduced (1) criteria to evaluate cell mergers from multiple vantage points and (2) methods to identify potential segmentation errors. Previous techniques only considered the forward projection of 2-D nuclei into neighboring z-slices and could not recognize 2-D segmentation errors, thus opening up the possibility for more errors with no means to identify them (Umesh Adiga and Chaudhuri, 1999). One novel feature of our 2-D to 3-D merging strategy is that it identifies both false positives (over-segmented objects) and false negatives (under-segmented objects) by evaluating cell mergers from multiple vantage points. Previous techniques have had to run multiple algorithms to perform certain cell mergers and have lacked user validation capabilities to eliminate their remaining errors ranging from 5 to 8% (Latorre et al., 2013).

In the future, we plan to implement improvements to the merging algorithm. While our algorithm identifies both false positives and false negatives, we have no method in place to distinguish between these error types, nor do we have routines to “split” under-segmented cells. Additionally, we only used two z-slices to determine if and how adjacent 2-D objects should be merged. In the future, it would be beneficial to use information from all overlapping 2-D objects (that typically traverse 3 or more z-slices) to arbitrate decisions about the error type (false positive, false negative) and the appropriate action (merge, split). One approach could use geometric statistics from local (only overlapping objects) and global (all 2-D objects) segmentation boundaries, along with an empirically derived cost function, to facilitate merging and splitting decisions. The method of applying a cost function to arbitrate merging decisions was demonstrated with great success (Lin et al., 2003). By integrating this approach into our merging routines, we could automatically correct over- *and* under-segmented somata, thereby requiring less software assisted manual input.

Future extensions of this software may prove useful in addressing challenging neural-specific image analysis applications where efficient and accurate automated routines are required. For instance, our 3-D context segmentation correction algorithms may improve the efficiency and accuracy of analyzing systems with increased densities of labeled cells (e.g., dense cortical regions in brain tissue or dense *in vitro* neural constructs) or the assessment of 3-D co-localization of multiple fluorescent labels. Additionally, although the presented methods were used exclusively to catalog information regarding somata, investigators may be able to utilize knowledge of cell body boundaries to facilitate additional morphological analyses, such as quantifying the spatial extent and volume of neurite outgrowth. Such detailed analyses based on cell geometry and connectivity are particularly relevant to neurobiological applications, yet are obviously not possible based solely on traditional nuclear staining. For instance, neuron-specific morphological-functional relationships have been noted previously, including that electrophysiological and/or membrane properties correlate with geometrical parameters such as volume and surface area. Additionally, increased somatic volume has been shown to correlate with increased dendritic complexity and axonal enlargement, while the number of synaptic sites remains fairly constant along dendrites and axons (Gutierrez-Ospina et al., 2004; Seeger et al., 2005). Thus, measurements of somatic/neuritic volume may correlate strongly with the number of synapses per neuron. Additionally, points of neuritic-neuritic or neuritic-somatic junctures may be identified morphologically as sites of potential synapses for network mapping applications.

The work presented here is an important step toward our long-term objective of developing algorithms that enable automated real-time analyses, thus incorporating temporal analyses over three spatial dimensions. Notably, we have recently begun utilizing the next generation of these techniques for functional neural network tracing by assessing the relative fluorescent intensity and signal propagation using voltage or ion-sensitive dyes in neural cultures. Clearly, for this application, data pertaining to full neuronal morphology and network connectivity across relevant spatial dimensions are crucial. Ultimately, with further development these techniques may lead to the ability for automated real-time 4-D analysis of ensemble electrophysiological network functionality and signal propagation across neural tissue or constructs.

### Conflict of interest statement

The authors declare that the research was conducted in the absence of any commercial or financial relationships that could be construed as a potential conflict of interest.
